# A Mobile Health App to Support Home-Based Aerobic Exercise in Neuromuscular Diseases: Usability Study

**DOI:** 10.2196/49808

**Published:** 2024-03-15

**Authors:** Tim Veneman, Fieke Sophia Koopman, Sander Oorschot, Pien G Koomen, Frans Nollet, Eric L Voorn

**Affiliations:** 1 Amsterdam University Medical Center location University of Amsterdam Rehabilitation Medicine Amsterdam Netherlands; 2 Amsterdam Movement Sciences, Rehabilitation & Development Amsterdam Netherlands

**Keywords:** neuromuscular disorders, endurance training, home-based exercise, eHealth, tele-rehabilitation, app, exercise, aerobic exercise, mhealth, mobile app, neuromuscular disease, usability

## Abstract

**Background:**

Home-based aerobic exercise in people with neuromuscular diseases (NMDs) has benefits compared to exercise in the hospital or a rehabilitation center because traveling is often cumbersome due to mobility limitations, and societal costs are lower. Barriers to home-based aerobic exercise include reduced possibilities for monitoring and lack of motivation. To overcome these and other barriers, we developed a mobile health app: Keep on training with ReVi (hereafter referred to as ReVi).

**Objective:**

We aimed to determine the usability of the ReVi app.

**Methods:**

Patients followed a 4-month, polarized, home-based aerobic exercise program on a cycle or rowing ergometer, with 2 low-intensity sessions and 1 high-intensity session per week supported by the ReVi app. The app collected training data, including heart rate and ratings of perceived exertion, provided real-time feedback on reaching target intensity zones, and enabled monitoring via an online dashboard. Physiotherapists instructed patients on how to use the ReVi app and supervised them during their training program. Patients and physiotherapists separately evaluated usability with self-developed questionnaires, including 9 questions on a 5-point Likert scale, covering the usability elements efficiency, effectiveness, and satisfaction.

**Results:**

Twenty-nine ambulatory adult patients (n=19 women; mean age 50.4, SD 14.2 years) with 11 different slowly progressive NMDs participated. Both patients and physiotherapists (n=10) reported that the app, in terms of its efficiency, was easy to use and had a rapid learning curve. Sixteen patients (55%) experienced 1 or more technical issue(s) during the course of the exercise program. In the context of effectiveness, 23 patients (81%) indicated that the app motivated them to complete the program and that it helped them to exercise within the target intensity zones. Most patients (n=19, 70%) and physiotherapists (n=6, 60%) were satisfied with the use of the app. The median attendance rate was 88% (IQR 63%-98%), with 76% (IQR 69%-82%) of time spent within the target intensity zones. Four adverse events were reported, 3 of which were resolved without discontinuation of the exercise program.

**Conclusions:**

The usability of the ReVi app was high, despite the technical issues that occurred. Further development of the app to resolve these issues is warranted before broader implementation into clinical practice.

## Introduction

Physical fitness is an important health marker [1,2] and is strongly associated with daily life functioning [3] and independent living [4] at older age. People with neuromuscular disease (NMD) often have reduced physical fitness caused not only by the underlying disease but also by an inactive lifestyle [5-7]. Aerobic exercise is an important aspect of rehabilitation treatment for NMD, as it contributes to improved physical fitness [8]. The integration of exercise programs into everyday life was recently identified as one of the major research priorities for individuals with NMD [9].

People with NMD usually perform their aerobic exercise program in a hospital, rehabilitation center, or physiotherapy practice under the direct supervision of a physiotherapist. However, center-based exercise may be cumbersome for individuals with NMD, who are often limited in their mobility. Moreover, center-based exercise requires the availability of physiotherapy staff, whose number is often limited as many countries are reducing health care services [10,11]. This amplifies the need for alternative modes of exercise intervention delivery that maintain high quality and effectiveness [12,13].

Transferring aerobic exercise from the hospital environment to the home or community may be a beneficial way to reduce travel time and societal costs. A recently developed training guide called B-FIT is an example of a home-based aerobic exercise program specifically developed for NMD [14]. Feasibility of the B-FIT exercise program has been demonstrated for different types of NMD, and patients and physiotherapists were satisfied with its use [14]. A barrier to use B-FIT was that some patients experienced the program as insufficiently challenging. This requires attention because poor motivation has been reported as a major barrier to exercise in people with NMD [7,15]. Furthermore, physiotherapists perceived initiation of the program as time-consuming; most of the worksheets, including exercise testing results and the training schedules, needed to be filled out by hand. A more general concern regarding exercise in the home environment is the reduced possibility to monitor exercise sessions. This is particularly important for the vulnerable population of people with NMD, as it may put them at risk for under- and overtraining.

To overcome these barriers to home-based aerobic exercise for people with NMD, we developed a mobile health (mHealth) app called Keep on training with ReVi (hereafter referred to as the ReVi app). The ReVi app aims to improve patients’ adherence to the B-FIT exercise program by (1) offering a structured exercise program, (2) providing insight into training progression, and (3) improving motivation through auditory encouragement. For physiotherapists, the ReVi app aims to improve their opportunities for supervision by enabling them to monitor progress and provide feedback from a distance and also to reduce the time investment to initiate the exercise program.

The primary aim of this study was to assess the usability of the ReVi app for assisting and monitoring home-based aerobic exercise according to the B-FIT training guide in people with NMD. We also evaluated the attendance rate, the time spent within target intensity zones, and the occurrence of adverse effects.

## Methods

### Design

A multicenter prospective pilot study was conducted at the outpatient departments of rehabilitation medicine of 2 university hospitals and in 3 rehabilitation centers in the Netherlands. All centers were specialized in treatment of NMD. This study included 2 different cohorts; in one cohort, the ReVi app was applied as part of usual care at the Department of Rehabilitation Medicine at the Amsterdam UMC, location Amsterdam Medical Center (AMC). The other cohort consisted of patients using the ReVi app in the intervention group of an ongoing multicenter randomized controlled trial on the efficacy of a physical activity program, which combines the B-FIT aerobic exercise program and motivational interviewing coaching to improve physical fitness in people with NMD [16].

### Ethical Considerations

The medical ethics review committee of the AMC waived the need for medical ethical approval for the usual care cohort, and approved the study protocol of the randomized controlled trial (NL62104.018.17). All patients provided informed consent.

### Participants

The inclusion criteria applied to both cohorts were (1) diagnosis of a slowly progressive NMD, (2) age ≥18 years, and (3) possession of a smartphone or tablet. Exclusion criteria were (1) contraindication for being physically active, (2) inability to follow verbal or written instructions, and (3) insufficient competence in the Dutch language. In addition, patients in the randomized controlled trial had to be motivated to improve their reduced physical fitness and were excluded if they had participated in an exercise program for a period longer than 4 weeks in the past 6 months. For the purpose of this study, we included only data of patients who completed at least 12 of the 48 possible training sessions, to ensure sufficient experience with the use of the ReVi app to evaluate its usability. We aimed to include a total of 30 patients in this study.

Physiotherapists were included in the study if they supervised at least 1 patient. Physiotherapists that were already exposed to the B-FIT training guide followed a half-day training course to refresh their knowledge on the use of the B-FIT training program and learn the use of the ReVi app. Physiotherapists that were not exposed to the B-FIT training guide followed a full-day training course to learn both the B-FIT training program and the use of the app. Furthermore, they received an instruction manual with a step-by-step guide on the use of the app.

### ReVi App

The ReVi app (Amsterdam UMC) was built by a company (everywhereIM BV) specialized in the development of medical apps. The app was available for iOS and Android and it was developed in the Dutch language. An expert group consisting of physiotherapists, rehabilitation physicians, exercise physiologists, patients with different types of NMD, and representatives of the Dutch Society of Muscle Diseases and of the app builder actively participated in the development of the ReVi app. Expert group meetings were organized to discuss the aims of the app, to identify essential functionalities, and to provide feedback on so-called functional designs (on paper). The primary objective during this initial developmental phase was to create an app to assist a 16-week aerobic exercise regimen. If the study yields favorable results, the next developmental stage will be initiated to enhance the app’s functionality and further explore the possibilities for offering longer-term support to home-based aerobic exercise. The data protection officer of Amsterdam UMC (location AMC) was also involved in the app’s development process to ensure that personal data processing was organized in accordance with the General Data Protection Regulation (GDPR).

#### B-FIT Aerobic Exercise Program

The ReVi app was programmed with the B-FIT aerobic exercise program. This 16-week, polarized, home-based exercise program consisted of 2 low-intensity sessions below the anaerobic threshold (AT) and 1 high-intensity session above the AT per week. Patients visited the study center prior to the start, midway through, and after completion of the exercise program for a face-to-face meeting with their supervising physiotherapist. During each visit, an exercise test was executed. During the visits midway through and after completion of the exercise program, patients received feedback on training progress based on exercise testing results and based on data in the ReVi dashboard (see section App Description).

In the usual care cohort, target intensity zones were based on indirect assessment of the AT using ratings of perceived exertion (RPEs) during a submaximal exercise test [17]. In the randomized controlled trial cohort, target intensity zones were based on direct assessment of the AT during an exercise test through visual inspection of the gas exchange plots using the V-slope method [18]. If training based on heart rate was not feasible, for instance in patients using β-blocking agents, training was based on RPEs using the 6-20 Borg scale. Each training session consisted of several exercise intervals interspersed with recovery periods. Training sessions were performed in the home environment (eg, at home, in the gym, or at a physiotherapy practice) on a bicycle or rowing ergometer. A more detailed description of the B-FIT aerobic exercise program can be found in Multimedia Appendix 1 [14].

#### App Description

Physiotherapists created a personal account for a web-based dashboard that was used to create and manage ReVi app accounts of patients they supervised. The dashboard could be accessed using a desktop or laptop computer. Two-way verification using Google Authenticator (Google Inc) was required to sign in. The physiotherapists created patient accounts by sending a link to the patients’ email addresses. Via this link, a password was created. Patients used the ReVi app on a mobile phone or tablet. Logging in to the ReVi app required their personal email address and password.

**Figure 1:**
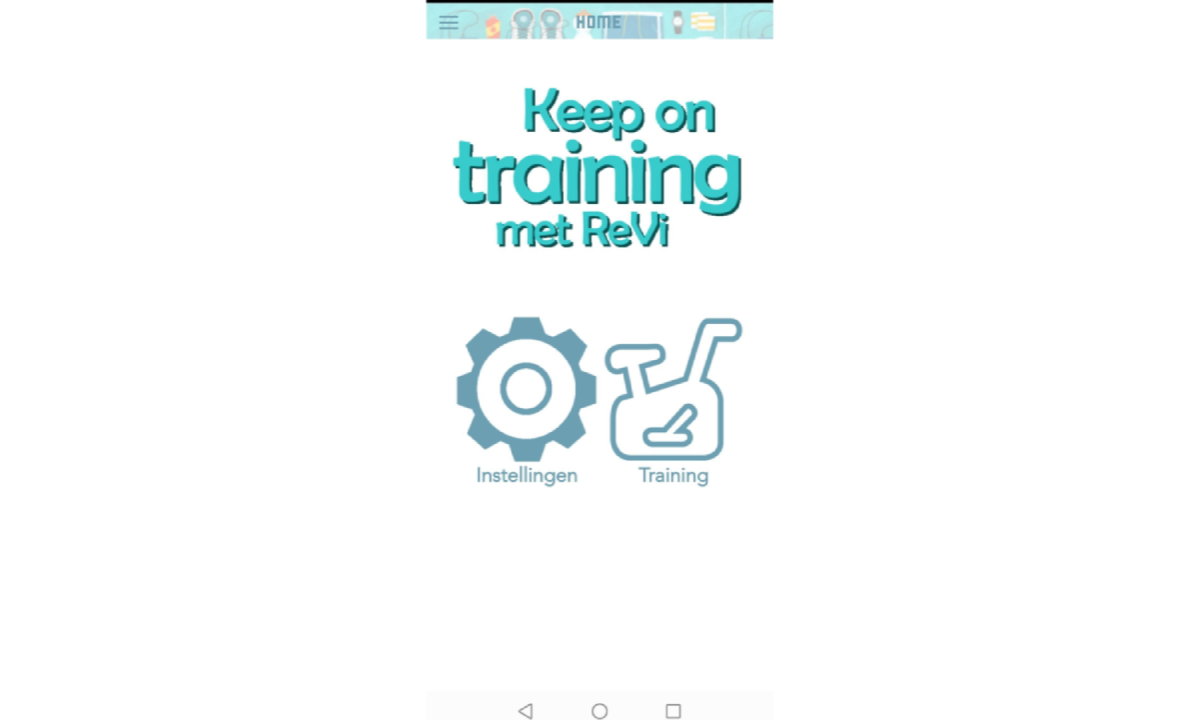
Screenshot of the ReVi app home screen.

After signing in to the ReVi app, the home menu opened, from which 2 menus could be chosen: the Settings menu and the Training menu, which provided an overview of the program (Figure 1). Through the Settings menu the type of training could be chosen: training based on heart rate or based on Borg scale. For training based on heart rate, a Bluetooth connection with a heart rate monitor was established (in these cohorts, the device was the Polar H10; Polar Electro) and could be tested. Additionally, contact details of the physiotherapist were entered to enable patients to contact their therapists via the ReVi app.

**Figure 2:**
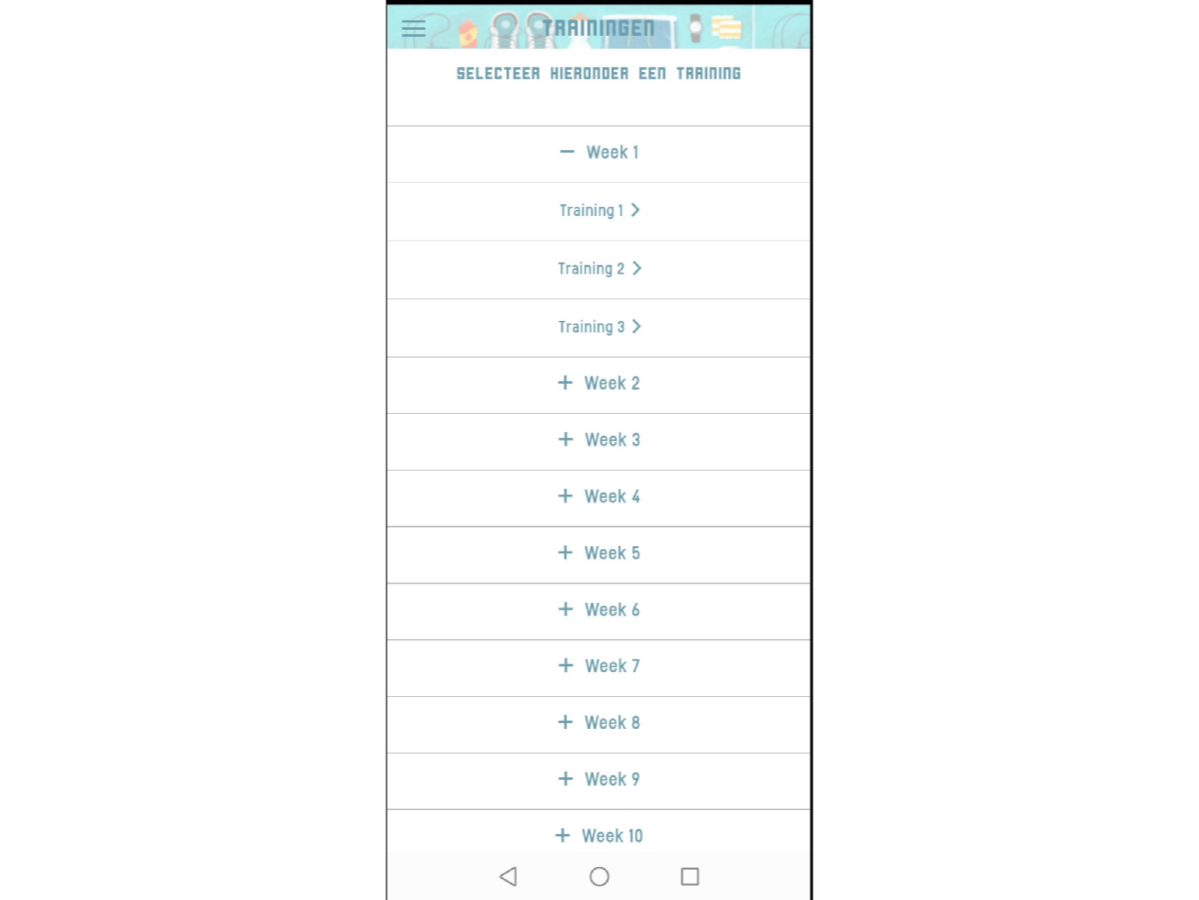
Screenshot of the exercise program overview.

**Figure 3:**
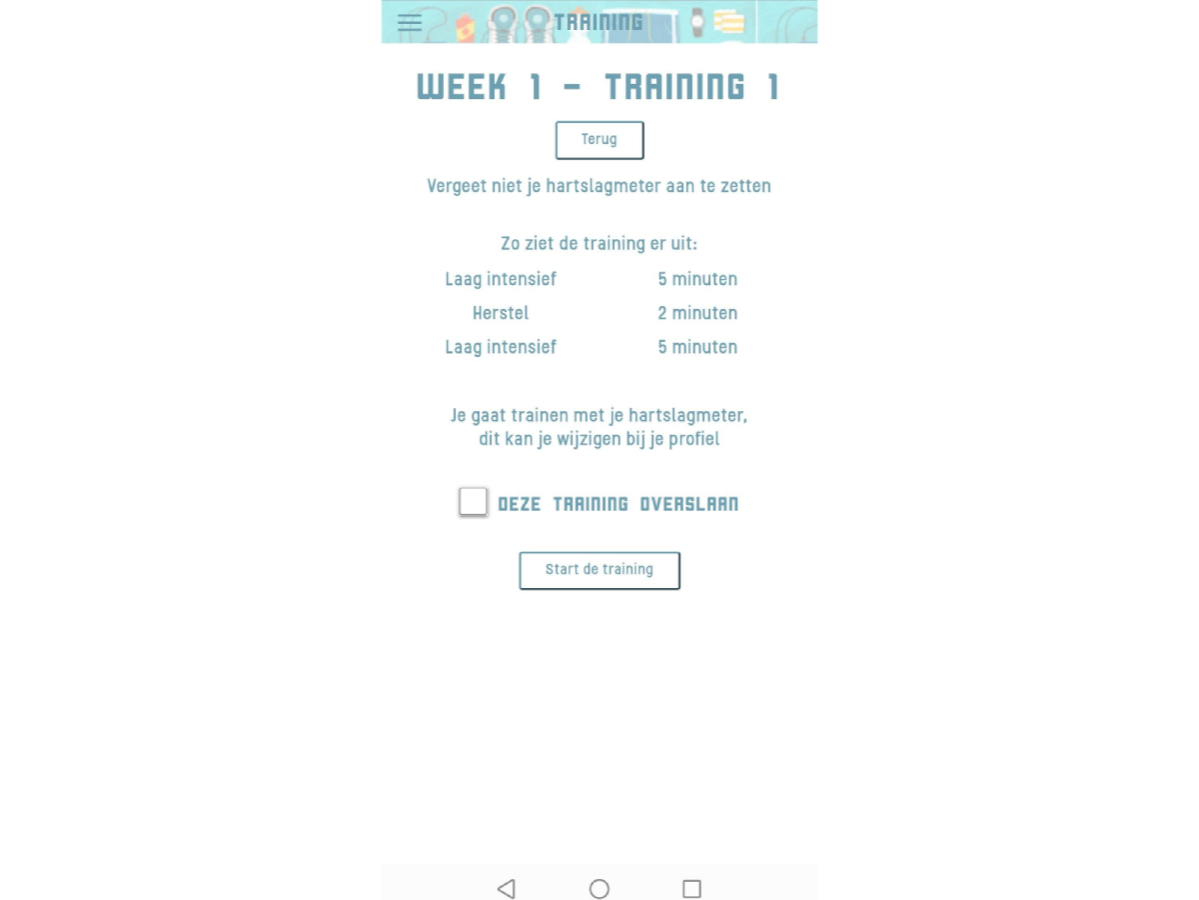
Screenshot of the exercise session protocol; this is an overview of the intensity and duration of each exercise or recovery bout.

**Figure 4:**
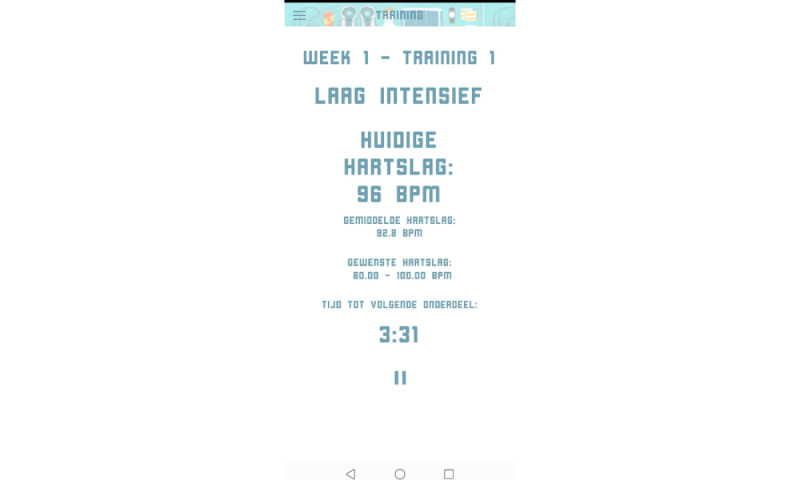
Screenshot of the exercise session live screen; the actual achieved intensity (heart rate or rating of perceived exertion) and the target intensity zone during the exercise session are shown.

**Figure 5:**
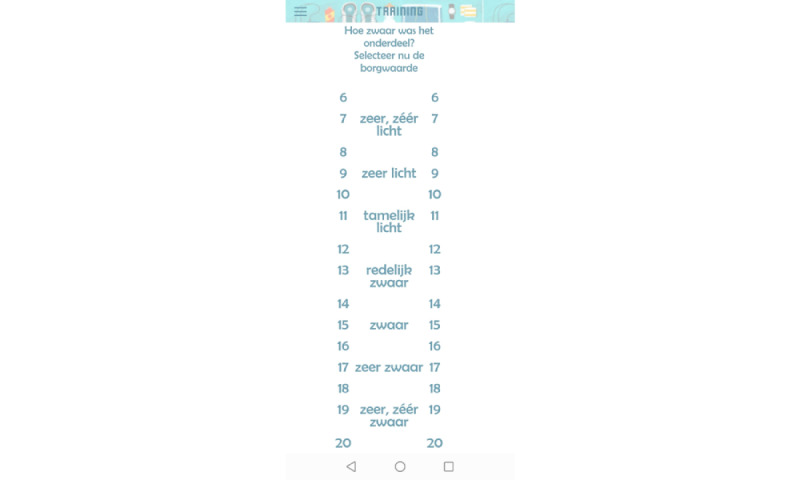
Screenshot of the Borg scale.

**Figure 6:**
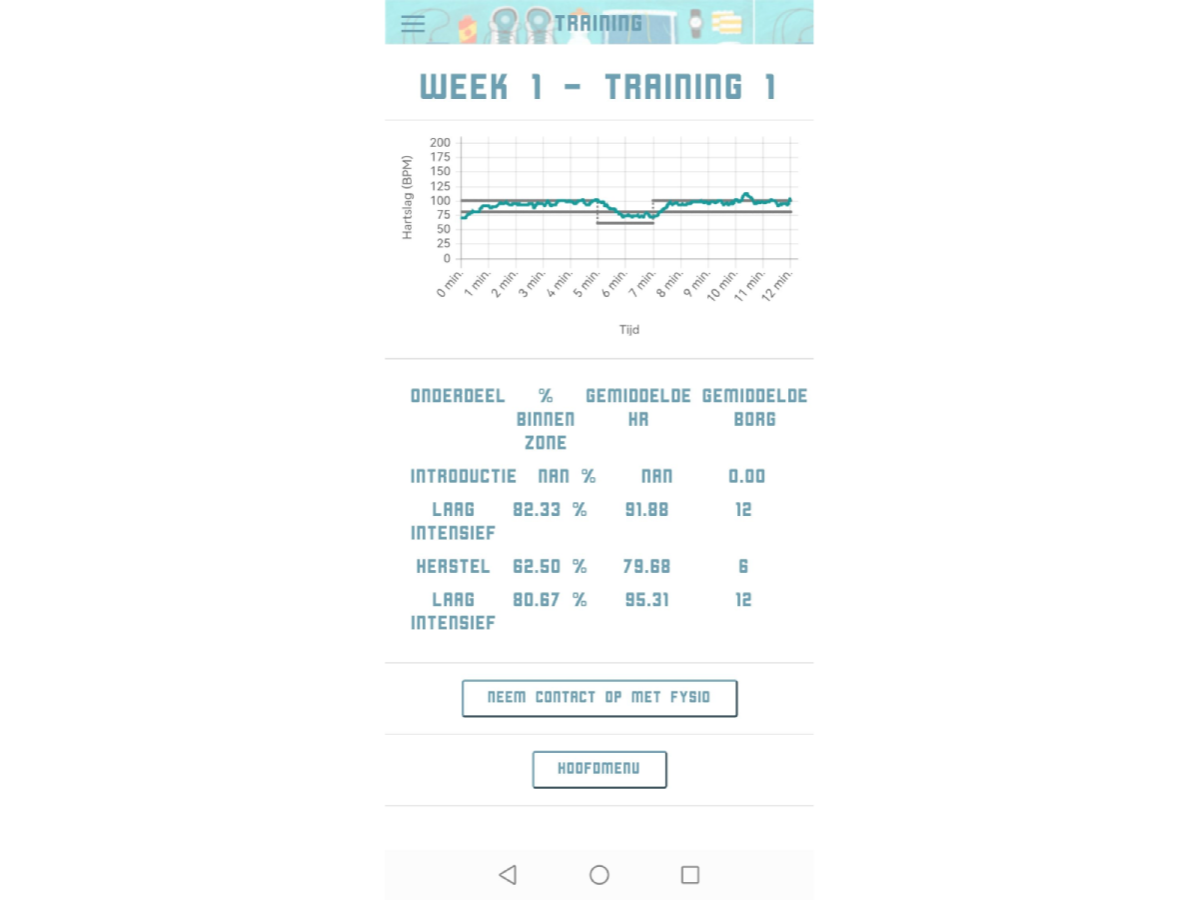
Screenshot of the exercise session results; the graph shows heart rate progression over time and the percentage of time spent within the target intensity zones.

The Training menu provided an overview of the training sessions (Figure 2). By selecting training sessions, the training protocol, including exercise intervals and recovery periods, was shown (Figure 3). During training sessions, the ReVi app guided users by illustrating their target intensity zones. In case of heart rate–based training, a heart rate chest strap was provided to the patient. The app was Bluetooth connected to the heart rate chest strap to continuously monitor heart rate (Figure 4). Patients rated their perceived exertion every final minute of the exercise interval or recovery period using the 6-20 Borg scale (Figure 5). During RPE-based training, patients rated their perceived exertion every minute. The ReVi app provided auditory feedback during training sessions. When patients trained within the target intensity zone, they were encouraged to continue. If the heart rate or Borg scale was not within the target intensity zone for at least 20 seconds, the ReVi app provided auditory instructions to increase or decrease the resistance. Directly after completion of the exercise session, an overview of the exercise results was shown (Figure 6).

**Figure 7:**
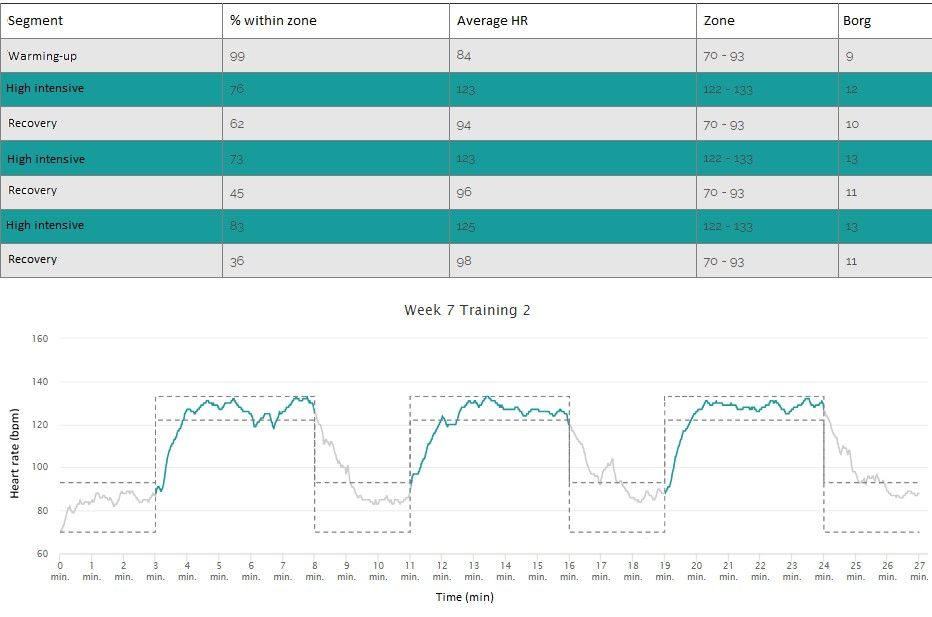
Screenshot of the ReVi app web-based dashboard. The percentage of time spent within the target intensity zones is presented in the table. The graph shows the heart rate during the training session (solid line), as well as the target intensity zones (grey dashed lines). The ReVi app dashboard is only available in Dutch, but for this paper the screenshot was translated to English. HR: heart rate.

Heart rate and Borg score data were saved by the ReVi app and sent to the web-based dashboard. Physiotherapists could access the training data of the patients they supervised; patients only had access to their own exercise data. The dashboard included, for each training session, a table with the percentage of time spent within the target intensity zones, the average heart rate for each exercise interval and recovery period, and the accompanying RPE (Figure 7). Additionally, a graph illustrated the actual heart rate or RPE with reference to the target intensity zones.

### Outcomes

#### ReVi App Usability

The primary outcome was the usability of the ReVi app, defined according to the International Organization for Standardization (ISO) as follows: “Usability is the extent to which a product can be used by specified users to achieve specified goals with efficiency, effectiveness and satisfaction in a specified context of use” [19]. Efficiency refers to the resources expended in relation to the accuracy and completeness with which users achieve goals (eg, ease of use, learning time, and additional effort of using the ReVi app during training sessions). Effectiveness refers to the extent to which the ReVi app has completed its goals to motivate patients and support patients to train within the targeted heart rate zones. Satisfaction assesses positive or negative attitudes toward the use of the ReVi app [20].

Self-developed questionnaires were used to assess the usability of the ReVi app among patients and physiotherapists. The questionnaires were developed by the study team, which consisted of researchers, rehabilitation physicians, and a physiotherapist. The questionnaires were reviewed by 2 patients and another physiotherapist before the final version was developed. The questionnaires contained questions pertaining to the 3 major aspects of usability: efficiency, effectiveness, and satisfaction. The usability questionnaires for patients and physiotherapists included 12 and 13 questions, respectively, of which 2 were open questions (Multimedia Appendices 2 and 3). Nine of the closed questions were scored on a 5-point Likert scale (1=strongly disagree; 2=disagree; 3=neither agree nor disagree; 4=agree; 5=strongly agree). Patients filled in the questionnaire after their last completed training session; physiotherapists did so after completion by the last patient they supervised.

#### Attendance Rate and Time Within Target Intensity Zones

For assessing attendance rates and the time spent within target intensity zones, we used data collected in the ReVi app dashboard. The attendance rate was defined as the percentage of followed training sessions. From the followed training sessions, we determined the percentage of time spent within target intensity zones for low- and high-intensity exercise intervals combined and separately.

#### Adverse Events

Adverse events related to the exercise program, such as severe muscle fatigue, joint pain, or muscle pain, were recorded. Patients were instructed to contact the physiotherapist to report adverse events. In addition, physiotherapists checked for adverse events during each patient visit.

### Data Analysis

Descriptive statistics are used to present patient and physiotherapist characteristics. The data from the questions that were scored on a 5-point Likert scale were reduced by combining “agree” and “strongly agree” responses to form an “agree” category, and response options of “strongly disagree” and “disagree” were combined to form “disagree.” Frequencies were calculated on the basis of the total number of responses to each question on the usability questionnaire and expressed as percentages. Data analysis was performed using SPSS (version 28.0; IBM Inc).

## Results

### Study Group

Between January 2020 and November 2021, 23 patients started their exercise program as part of the usual care cohort, of which 20 patients were included in the study. Three patients were excluded because they executed less than 12 exercise sessions. Reasons included technical problems with the ReVi app (n=1), medical issues (n=1), and a lack of motivation (n=1). Nine other patients participating in the ongoing randomized controlled trial were also included and started between July 2021 and December 2021.

Patient characteristics are shown in Table 1. Twenty-three patients were treated at the outpatient clinic of the Department of Rehabilitation of the Amsterdam UMC (location AMC), supervised by 6 physiotherapists. The other 6 patients were treated by 4 physiotherapists at Rehabilitation Center Klimmendaal (Arnhem; n=2), Basalt Rehabilitation Center (Leiden; n=2), University Medical Center Utrecht (Utrecht; n=1), and Sint Maartenskliniek (Nijmegen; n=1). Twenty-eight patients trained based on heart rate and 1 patient based on the Borg scale. Twenty-seven patients performed the exercise program using a bicycle ergometer and 2 patients used a rowing ergometer.

**Table 1:** Respondent profile.

Characteristics				Respondents
**Patients (n=29)**				
	Age (years), mean (SD)		50.4 (14.2)	
	Female, n (%)		19 (66)	
	Sum score of manual muscle testing for the legs^a^, median (range)		75 (60-80)	
	Peak workload baseline submaximal exercise test (watts), median (range)		100 (50-210)	
	**Types of neuromuscular disorder, n**			
		Charcot-Marie-Tooth disease	7	
		Myotonic dystrophy	4	
		Nonspecific myopathy	4	
		Congenital dystrophy	3	
		Limb girdle muscular dystrophy	3	
		Mitochondrial myopathy	2	
		Inclusion body myositis	2	
		Becker muscular dystrophy	1	
		Postpolio syndrome	1	
		Dermatomyositis	1	
		Chronic inflammatory demyelinating polyradiculoneuropathy	1	
**Physiotherapists (n=10)**				
	Female, n (%)		7 (70)	
	Patients supervised in study (n), median (range)		2 (1-9)	
	Prior experience with use of the ReVi app, n (%)		4 (40)	

^a^Sum score for muscle strength of the legs was calculated by adding 16 muscle groups. Each muscle group had a score between 0 and 5, and the sum score ranged from 0 to 80 [21].

### Primary Outcome

#### Usability

**Figure 8:**
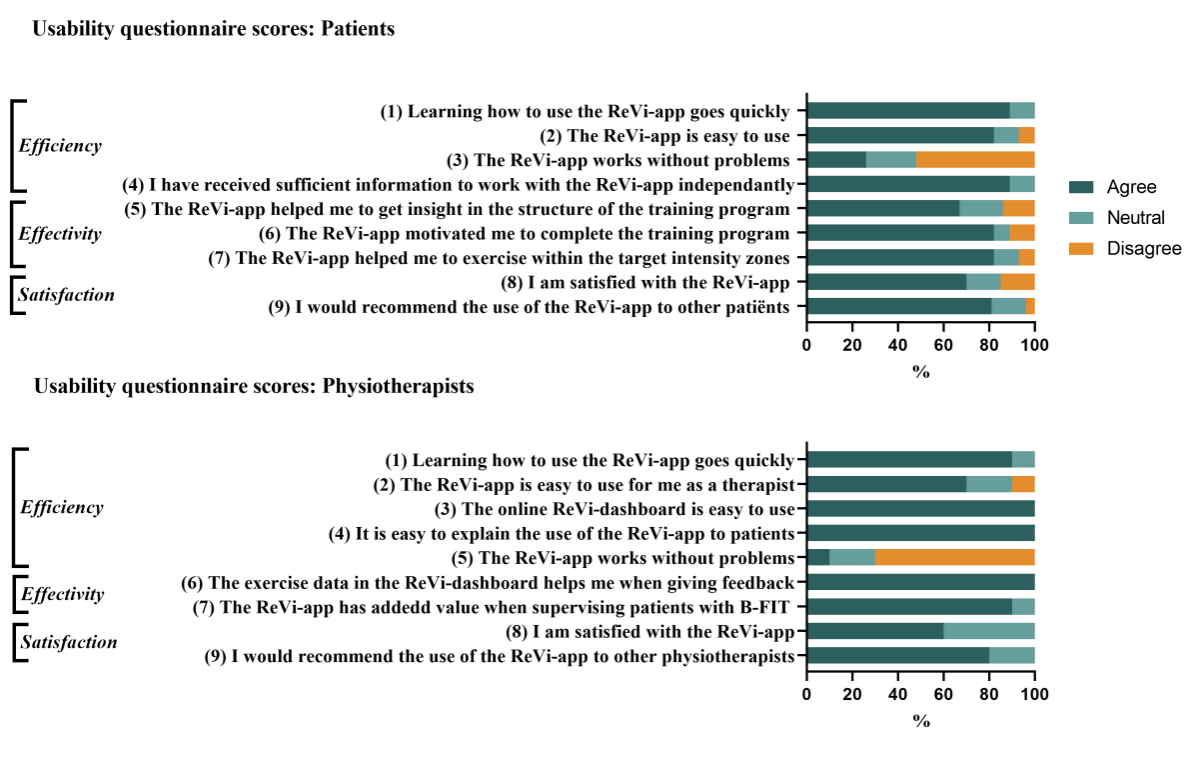
Statements and outcomes for the patient usability questionnaire (n=27) and the physiotherapist usability questionnaire (n=10). Scores are given on a Likert scale, ranging from 1-5 (1 = strongly disagree, 5 = strongly agree). Frequency data were reduced by combining “agree” and “ strongly agree” responses to form an “agree” category, and response options of “strongly disagree” and “disagree” were combined to form “disagree”.

Twenty-seven patients and all 10 physiotherapists filled in and returned the usability questionnaire. Two patients did not return the usability questionnaire despite multiple requests. Questionnaire scores of patients and physiotherapists are presented in Figure 8.

#### Efficiency

Twenty-four patients (89%) reported that learning how to use the ReVi app went quickly and 22 patients (81%) found that the ReVi app was easy to use. Seven patients (26%) agreed with the statement “the ReVi app works without problems.” In 16 of the total of 29 patients (55%), 1 or more technical issues occurred during the course of the ReVi app training program. The most-reported technical issues were connection problems with the heart rate monitor and a bug in the app that hindered saving of exercise data in week 11 of the exercise program.

The majority of therapists reported that the ReVi app was easy to use (n=7, 70%) and all therapists found the use of the app easy to explain to patients. Nine therapists (90%) experienced technical issues using the ReVi app.

#### Effectiveness

Eighteen patients (67%) reported that the ReVi app provided insight into the structure of the exercise program. Twenty-two patients (81%) agreed that the app motivated them to complete the program and that it helped them to maintain exercise within the target intensity zones.

All therapists reported that the web-based dashboard helped them to provide feedback to patients and that the ReVi app had added value for supervision. The most important benefit reported by the physiotherapists was that the ReVi app allowed insight into the number of sessions that were followed and the exercise intensity that was achieved during training sessions.

#### Satisfaction

Nineteen patients (70%) were satisfied with the use of the ReVi app and 22 patients (81%) would recommend the use of the ReVi app to other patients with NMD. The most important reasons to recommend its use to others were that the app provided structure, helped them to train within the target intensity zones, and motivated them to complete their training sessions. The most-reported reason for patients not to recommend the ReVi app to others was the occurrence of technical issues.

Six therapists (60%) reported that they were satisfied with the use of the ReVi app and 8 therapists (80%) would recommend the use of the app to other physiotherapists. Reasons to recommend its use to others were that it was easy to use, enabled monitoring from a distance, and provided data that could be used to give tailored feedback to patients. The most-reported reason to not recommend the ReVi app to others was the technical issues that occasionally occurred when using the app.

### Secondary Outcomes

#### Attendance and Time Within Target Intensity Zones

Twenty of the 29 patients (69%) completed the exercise program. Reasons for discontinuation among the other 9 patients were technical problems with the ReVi app (n=4), medical issues (n=2), closing of the local gym due to COVID-19 measures (n=2), and a lack of motivation (n=1).

**Figure 9:**
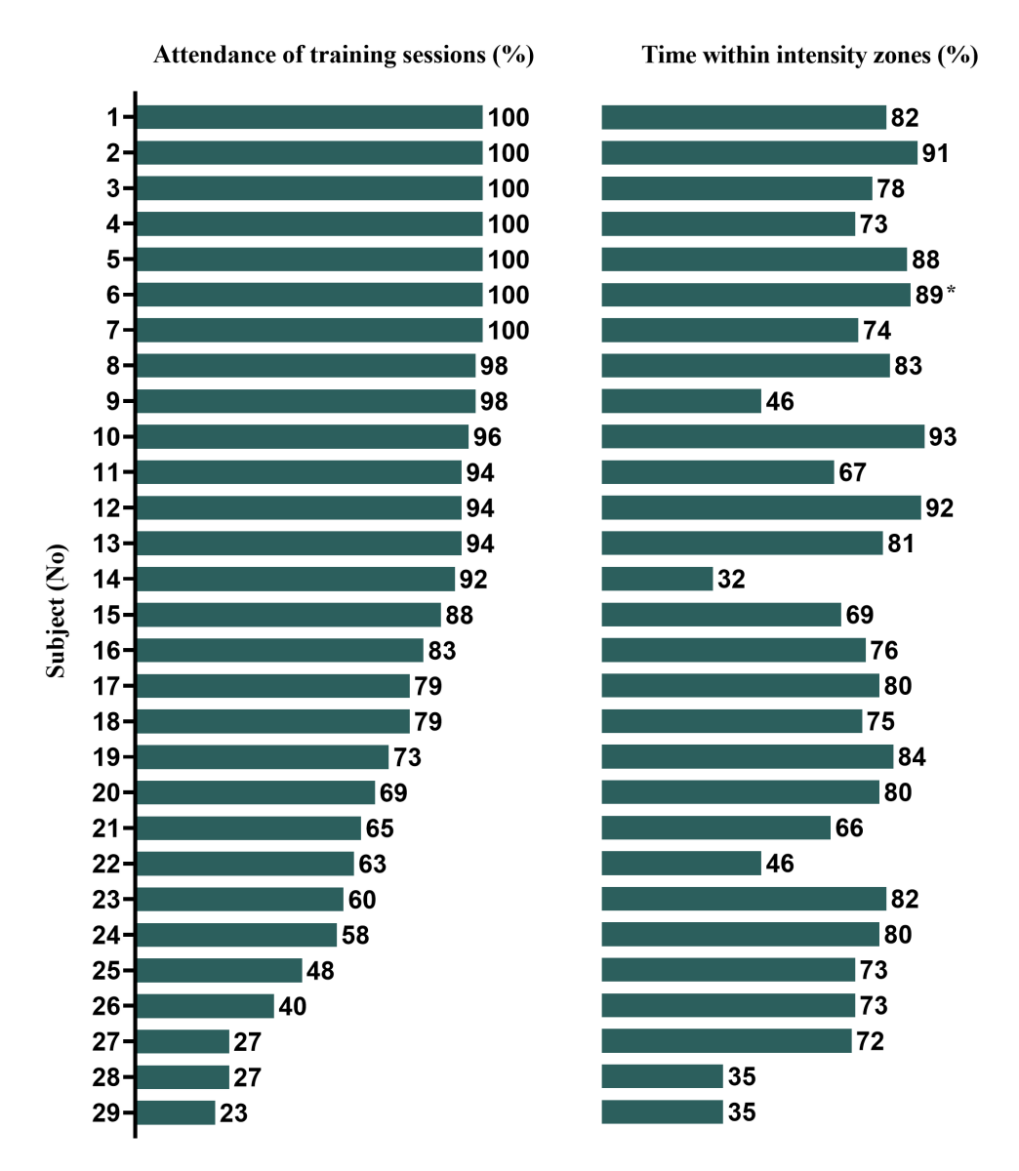
Attendance rates for individual patients ordered from most to least sessions, and the percentage of time spent within the target intensity zones during corresponding sessions. * patient trained based on Borg scale.

**Figure 10:**
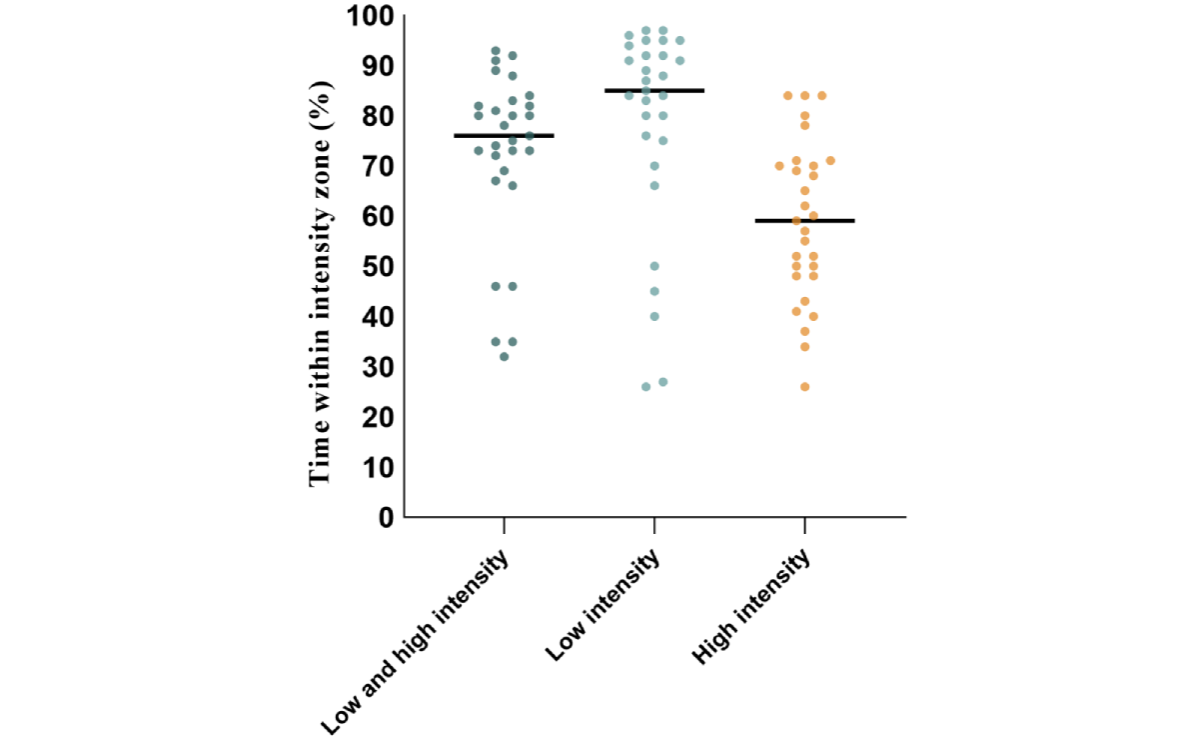
The percentage of time spent within the target intensity zones during A) both high and low intensity exercise intervals, B) low intensity exercise intervals and C) high intensity exercise intervals. Black lines indicate the median. Each dot represents a single patient.

Figure 9 shows the attendance rate for each patient, as well as the time spent within the target intensity zones. The median attendance rate was 88% (IQR 63%-98%). During the attended training sessions, patients spent a median of 76% (IQR 69%-82%) of the time within their target intensity zones (Figure 10). The median percentage of time spent within the low intensity zones was 85% (IQR 81%-92%), and in the high intensity zones it was 59% (IQR 45%-70%).

#### Adverse Events

Four adverse events were reported: fatigue (n=2), knee joint pain (n=1), and high blood pressure during training (n=1). In the patient with high blood pressure during training, the rehabilitation physician and physiotherapist decided to terminate the exercise program. The other 3 adverse events were resolved without discontinuation of the exercise program.

## Discussion

### Principal Findings

This study provides insight into the usability of the ReVi app among people with NMD to support home-based aerobic exercise according to the B-FIT training program. The different components of usability, including efficiency, effectiveness, and satisfaction, were all judged as good by physiotherapists and patients, despite the occurrence of technical issues.

Patients were generally positive about the efficiency of the ReVi app due to its rapid learning curve and ease of use. Patients could independently work with the app based on the instructions that they received from their treating physiotherapist. Adequate instructions are known to be a key facilitator of patient engagement with mHealth apps [22]. With regards to its effectiveness, patients reported that the most important goals of the ReVi app were achieved: its use motivated them to complete the exercise program and helped them to exercise within their target intensity zones. These outcomes were supported by the findings that patients attended the majority of training sessions and spent most time within the target intensity zones. Patients were mostly satisfied with the use of the app, which concurs with other studies on apps supporting home-based physical exercise programs in amyotrophic lateral sclerosis, which is a rapidly progressive type of NMD [23], and a variety of other patient populations [24-26].

Physiotherapists were positive about the efficiency of the ReVi app. This was mainly due to the rapid learning curve and its ease of use; a half- or full-day training course was required for physiotherapists to learn how to work with the app and the B-FIT training guide, depending on prior experience with B-FIT. In terms of effectiveness, physiotherapists reported that the most important goals of the ReVi app were achieved. They found the app helpful when monitoring patients during their home-based program, mainly because it enabled them to provide feedback based on exercise data. They were generally satisfied with the use of the ReVi app and would recommend the use of the app to other physiotherapists.

While efficiency, effectiveness, and user satisfaction were overall judged as positive, one of the efficiency items was clearly judged as insufficient: 55% (n=16) of the patients and 90% (n=9) of the physiotherapists experienced technical issues. Most of these issues were solved, but in all cases, this required the help of a physiotherapist, researcher, or software developer. Technical issues are known to negatively impact usability and decrease adherence and engagement with mHealth tools [22]. They often cause patients to stop their mHealth interventions, leading to high dropout rates, and they are reported as a main barrier to further implementation of mHealth or eHealth apps [22,27]. This is consistent with our finding that the most important reason for discontinuation of the exercise program was when the ReVi app did not function well. Therefore, resolving technical issues is an important concern for further implementation of the ReVi app in clinical rehabilitation practice on a broader scale. This also underlines the importance of offering technical support when using mHealth tools such as the ReVi app [28]. Physiotherapists could play an important role in this, but that would require sufficient proficiency with mHealth. Moreover, considering the limited availability of physiotherapy personnel, it is essential for successful implementation of mHealth tools like the ReVi app to minimize technical issues and provide access to additional technical support for more complex problems.

The attendance rate, time within target intensity zones, and adverse events found in this study suggest that training in the home environment with the help of the ReVi app is a good alternative to center-based training. The attendance rate of 88% and time within target intensity zones of 76% are in line with adherence rates found in other studies evaluating aerobic exercise programs for NMD [29-37] that were mostly conducted in a hospital or rehabilitation center. Comparison of the attendance rate and time within target intensity zones between this study and past studies on exercise for NMD is hampered by incomplete or absent descriptions of adherence assessment methods in most other studies. In some studies, it is unclear if reported values are for attendance rates, the time spent within exercise zones, or training time. Moreover, some studies excluded patients who dropped out, leading to overestimated adherence. In this light, the attendance rate in our study may have been impacted by excluding patients who performed less than 12 exercise sessions and by the finding that some patients performed several training sessions without using the ReVi app. Despite these uncertainties, the attendance rate and time within target intensity zones found in our study seem to be in line with values reported in other aerobic exercise studies. The limited number of adverse events reported in this study also concurs with other studies on center-based aerobic exercise programs for NMD [32,38,39]. This further strengthens the notion that home-based aerobic exercise supported by the ReVi app may be considered a safe and feasible alternative for center-based exercise programs, which is in line with earlier research in telemonitoring of home-based exercise for amyotrophic lateral sclerosis [23].

### Limitations

Patients with a positive attitude toward the use of mHealth may have been more inclined to participate in this study, causing selection bias and limiting generalizability to people with NMD and less affinity for mHealth. Also, most patients trained under supervision of a physiotherapist experienced in treating patients with NMD, which limits generalizability of our results to other health care settings, such as primary care physiotherapy practices.

### Future Studies

Implementation of mHealth, such as with the ReVi app, in rehabilitation care presents some major challenges, such as the comfort of patients and therapists with the use of technology, legal and ethical considerations regarding patient monitoring and the protection of privacy rights, and integration of mHealth tools into current working protocols [40,41]. Additionally, specific application design requirements have to be considered for NMD patients who experience reduced hand functionality due to muscle weakness. These requirements may include sufficiently large buttons and input fields. As a consequence of these challenges, the scientific literature on telerehabilitation in NMD patients is still limited [42,43]. To enable the broader implementation of mHealth in clinical practice, research is warranted into other facilitators of and barriers to the implementation of mHealth specific to neuromuscular rehabilitation.

### Conclusions

The usability of the ReVi app in terms of perceived efficiency, effectiveness, and user satisfaction is high, despite the occurrence of technical issues. Combined with the high attendance rate and time spent within target intensity zones and low number of adverse events, the ReVi app can be considered a promising tool to support home-based aerobic exercise in rehabilitation practice for NMD. Further development of the ReVi app to resolve technical issues is warranted before broader implementation into clinical rehabilitation practice.

## Appendix

Multimedia Appendix 1 B-FIT exercise program.

Multimedia Appendix 2 Patient usability questionnaire.

Multimedia Appendix 3 Therapist usability questionnaire.

## Abbreviations

AMC: Amsterdam Medical Center
AT: anaerobic threshold
GDPR: General Data Protection Regulation
ISO: International Organization for Standardization
mHealth: mobile health
NMD: neuromuscular disease
RPE: rating of perceived exertion
