# Supplementary material for: A Mobile Health App to Support Home-Based Aerobic Exercise in Neuromuscular Diseases: Usability Study
Source: JMIR Hum Factors. 2024 Mar 15;11:e49808. doi: 10.2196/49808 (PMC10980987; doi:10.2196/49808)
Supplement: Multimedia Appendix 1 [file humanfactors_v11i1e49808_app1.docx]

**Appendix 1:** B-FIT exercise program

**Aerobic exercise intervention**

The aerobic exercise intervention consisted of a 4-month, polarized, home-based program, with 2 low-intensity sessions (ie, below the AT) and 1 high-intensity session (ie, above the AT) per week. Each training session consisted of 2 or 3 exercise bouts interspersed with 5- or 3-minute recovery periods, for low and high intensity, respectively. High-intensity training sessions were always preceded by a 3-minute warming up, and followed by a 3-minute cooling down. The duration was gradually increased from 10 to 17 minutes per bout for low-intensity sessions and from 3 to 8 minutes per bout for high-intensity sessions (Figure 1). This resulted in approximately 75% of the total training volume being performed at low intensities and approximately 25% at high intensities. Training sessions were performed in the home environment (eg, at home or in the local gym), preferably on a stationary ergometer, and the exercise program was coordinated and supervised by trained physical therapists experienced in treating patients with NMD.

Participants were supplied with a logbook containing training instructions to register the number of training sessions followed, their heart rate and perceived exertion on the Borg Scale (range, 6–20), and any physical complaints they experienced during or after training. Participants were responsible themselves for taking care of the necessary equipment (ie, heart rate monitor and ergometer) and facilities (eg, local gym, physical therapist practice).

**Incremental Submaximal Exercise Test**

Participants performed an incremental submaximal exercise test, which previously showed to be feasible in PPS and could be used to determine the AT. The test was supervised by the treating physical therapist and performed before, midway and directly after the exercise intervention period. The test was executed on a bicycle ergometer, or on a treadmill in case of a walking program. The exercise test consisted of 3 minutes unloaded cycling (or walking at 2 km/h), after which the workload was increased by 5 to 10 W (0.5–1.0 km/h in case of walking) every minute, depending on the patients’ physical capacity. Criteria for stopping the test were the heart rate exceeding 80% of the estimated HRR, the pedal frequency dropping below 60 revolutions per minute, or the participant being unable to continue the test for any reason. Heart rate was monitored throughout the test with a heart rate monitor. Midway each workload and at the end of the exercise test, participants rated their perceived exertion (RPE) on the Borg Scale (range, 6–20), and the physical therapist registered this on a worksheet together with the heart rate.

**Target Intensity Zones**

Target intensity zones for the aerobic exercise program were based on the AT, which was determined from the submaximal exercise test. The AT was indirectly assessed by determining the heart rate corresponding to an RPE of 12 on the Borg Scale. A previous study in PPS demonstrated a high correlation (r = 0.86) with the AT. The preferred way of training was based on heart rate, but if necessary (eg, for participants using beta-blocking agents), training could also be based on the RPE using the Borg Scale (range, 6–20).


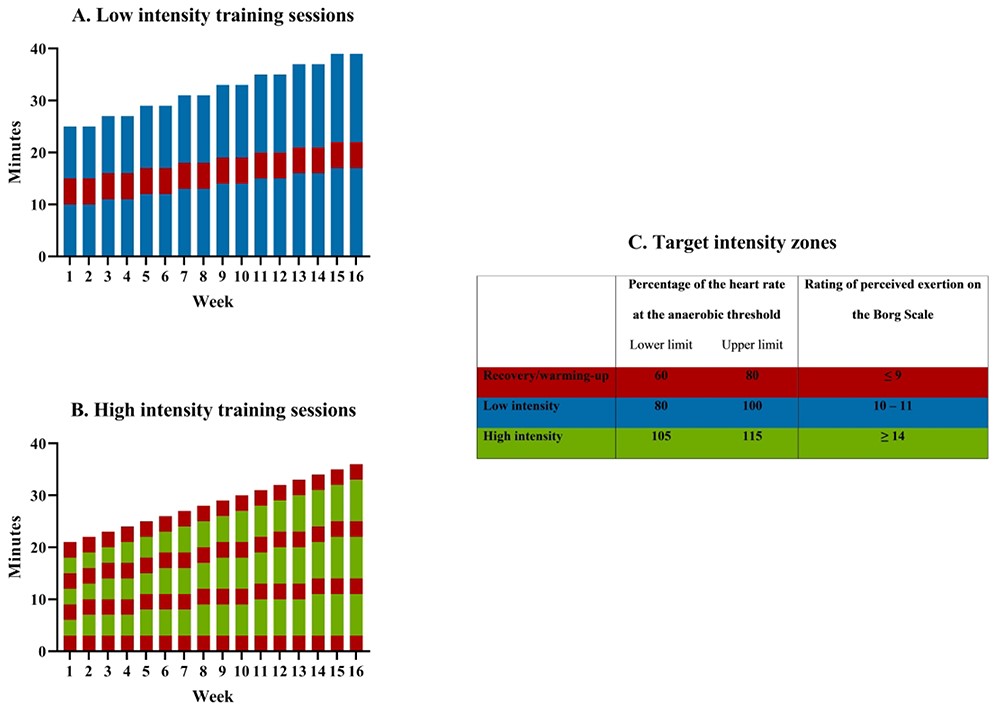


Figure 1: Overview of the B-FIT exercise program. Low-intensity training sessions (twice weekly) consisted of 2 exercise bouts interspersed with 5-minute recovery periods (A). High-intensity training sessions (once weekly) consisted of 3 exercise bouts interspersed with 3-minute recovery periods. High-intensity sessions were preceded by a 3-minute warming-up and followed by a 3-minute cooling down (B). The lower and upper limits of the target intensity zones were calculated using the heart rate at the anaerobic threshold as determined from submaximal exercise testing as a reference value (100%) (C).

**Reference**

Voorn EL, Koopman FS, Nollet F, Brehm M-A. Individualized Aerobic Exercise in Neuromuscular Diseases: A Pilot Study on the Feasibility and Preliminary Effectiveness to Improve Physical Fitness. Physical Therapy. 2021;101(3):pzaa213.
