# Supplementary material for: A Mobile Health App to Support Home-Based Aerobic Exercise in Neuromuscular Diseases: Usability Study
Source: JMIR Hum Factors. 2024 Mar 15;11:e49808. doi: 10.2196/49808 (PMC10980987; doi:10.2196/49808)
Supplement: Multimedia Appendix 2 [file humanfactors_v11i1e49808_app2.docx]

**Appendix 2:** Patient usability questionnaire

| **Patient questionnaire on the satisfaction with the use of the ‘Keep on training with ReVi’ app** |
| --- |

***Instructions***

During the past few months you have followed a physical training program. During this program you have used the *‘Keep on training with ReVi’* app, from now on in this questionnaire referred to as ‘ReVi-app’. We would like to evaluate your satisfaction with regard to the use of the ReVi-app, so that we can further develop the app. Therefore we ask you to fill out this questionnaire. This will take approximately 15 minutes.

Some of the questions may seem quite similar, but we ask you kindly to answer all the questions, because this provides us with important information for the further development of the training guide.

It is important that you give only 1 answer per question, namely the answer that is, according to you, most suited to your situation.

**Thank you for your cooperation!**


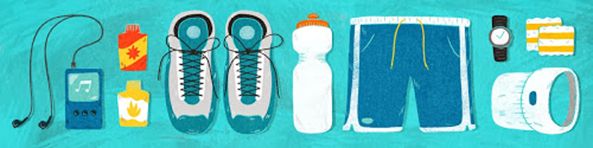


**1. Learning to work with the ReVi-app goes fast.**

Ο Strongly agree

Ο Agree

Ο Neutral

Ο Disagree

Ο Strongly disagree

**2. The ReVi-app is simple to use.**

Ο Strongly agree

Ο Agree

Ο Neutral

Ο Disagree

Ο Strongly disagree

**3. The ReVi-app works without problems.**

Ο Strongly agree

Ο Agree

Ο Neutral

Ο Disagree

Ο Strongly disagree

**4.** **I have received sufficient information from the physiotherapist to work with the ReVi-app independantly.**

Ο Strongly agree

Ο Agree

Ο Neutral

Ο Disagree

Ο Strongly disagree

**5. The ReVi-app helped me to gain insight into the structure of the exercise program.**

Ο Strongly agree

Ο Agree

Ο Neutral

Ο Disagree

Ο Strongly disagree

**6. The ReVi-app motivated me to maintain and finish the exercise program.**

Ο Strongly agree

Ο Agree

Ο Neutral

Ο Disagree

Ο Strongly disagree

**7. The ReVi-app helped me to exercise within the heart rate zones.**

Ο Strongly agree

Ο Agree

Ο Neutral

Ο Disagree

Ο Strongly disagree

**8. I have contacted the physiotherapist using the ReVi-app.**

Ο Yes

Ο No

**If ‘yes’:**

**How many times did you contact the physiotherapist using the ReVi-app?**

……………………………………………………………………………

**9. I am satisfied with the ReVi-app.**

Ο Strongly agree

Ο Agree

Ο Neutral

Ο Disagree

Ο Strongly disagree

**10. I would recommend other people with a neuromuscular disease that follow the B-FIT exercise program to use the ReVi-app.**

Ο Strongly agree

Ο Agree

Ο Neutral

Ο Disagree

Ο Strongly disagree

**11. Can you give reasons why you would recommend the ReVi-app to others?**

1…………………………………………………………………………………………………

2…………………………………………………………………………………………………

3…………………………………………………………………………………………………

**12. Can you give reasons why you would not recommend the ReVi-app to others?**

1……………………………………………………………………………………………………

2……………………………………………………………………………………………………

3……………………………………………………………………………………………………
