# Supplementary material for: A Mobile Health App to Support Home-Based Aerobic Exercise in Neuromuscular Diseases: Usability Study
Source: JMIR Hum Factors. 2024 Mar 15;11:e49808. doi: 10.2196/49808 (PMC10980987; doi:10.2196/49808)
Supplement: Multimedia Appendix 3 [file humanfactors_v11i1e49808_app3.docx]

**Appendix 3:** Therapist usability questionnaire

| **Therapist questionnaire on the satisfaction with the use of the ‘Keep on training with ReVi’ app** |
| --- |

***Instructions***

During the past period you have prescribed physical training programs (B-FIT) to patients. Hereby you have used the *‘Keep on training with ReVi’* app, from now on in this questionnaire referred to as ‘ReVi-app’. We would like to evaluate your satisfaction with regard to the use of the ReVi-app, so that we can further develop the app. Therefore we ask you to fill out this questionnaire. This will take approximately 15 minutes.

Some of the questions may seem quite similar, but we ask you kindly to answer all the questions, because this provides us with important information for the further development of the training guide.

It is important that you give only 1 answer per question, namely the answer that is, according to you, most suited to your situation.

**Thank you for your cooperation!**


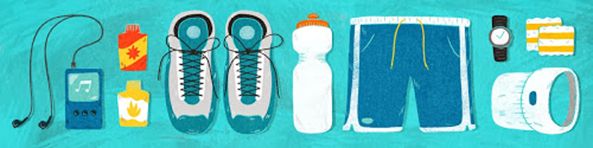


**1. Number of patients treated within study.**

Answer:……………………………………………………………………………………

**2. Do you have additional experience with supervising patients with the B-FIT exercise program, besides this study?**

Ο Yes

Ο No

**If ‘yes’:**

**How many patients did you supervise in total, outside of this study, begeleid bij het uitvoeren van het B-FIT programma?**

Answer:……………………………………………………………………………………

**3. Learning to work with the ReVi-app goes quickly.**

Ο Strongly agree

Ο Agree

Ο Neutral

Ο Disagree

Ο Strongly disagree

**4. The ReVi-app is simple to use for me as a therapist.**

Ο Strongly agree

Ο Agree

Ο Neutral

Ο Disagree

Ο Strongly disagree

**5. The online dashboard is simple to use for me as a therapist.**

Ο Strongly agree

Ο Agree

Ο Neutral

Ο Disagree

Ο Strongly disagree

**6. It is easy to explain the use of the ReVi-app to patients.**

Ο Strongly agree

Ο Agree

Ο Neutral

Ο Disagree

Ο Strongly disagree

**7. The ReVi-app works without problems.**

Ο Strongly agree

Ο Agree

Ο Neutral

Ο Disagree

Ο Strongly disagree

**8. The exercise data in the online dashboard of the ReVi-app help me when giving feedback to patients about their training progress.**

Ο Strongly agree

Ο Agree

Ο Neutral

Ο Disagree

Ο Strongly disagree

**9. The ReVi-app is of added value when supervising patients with the B-FIT exercise program.**

Ο Strongly agree

Ο Agree

Ο Neutral

Ο Disagree

Ο Strongly disagree

**10. I am satisfied with the ReVi-app.**

Ο Strongly agree

Ο Agree

Ο Neutral

Ο Disagree

Ο Strongly disagree

**11. I would recommend the ReVi-app to other physiotherapists who prescribe the B-FIT exercise program to patients.**

Ο Strongly agree

Ο Agree

Ο Neutral

Ο Disagree

Ο Strongly disagree

**12. Can you give reasons why you would recommend the ReVi-app to other physiotherapists?**

1…………………………………………………………………………………………………

2…………………………………………………………………………………………………

3…………………………………………………………………………………………………

**13. Can you give reasons why you would not recommend the ReVi-app to other physiotherapists?**

1……………………………………………………………………………………………………

2…………………………………………………………………………………………………

3…………………………………………………………………………………………………
